# Stabilizing Metastable Rare-Earth Ferrites on (111) Platinum via an Iron Oxide Interlayer

**DOI:** 10.1021/acsaelm.5c02616

**Published:** 2026-03-12

**Authors:** Marshall B. Frye, Jonathan R. Chin, Nicholas A. Parker, Steven E. Zeltmann, Matthew R. Barone, Darrell G. Schlom, Lauren M. Garten

**Affiliations:** † School of Materials Science and Engineering, 1372 Georgia Institute of Technology, Atlanta 30332, Georgia; ‡ Department of Materials Science and Engineering, 5922 Cornell University, Ithaca, New York 14853, United States; § Platform for the Accelerated Realization, Analysis, and Discovery of Interface Materials (PARADIM), Cornell University, Ithaca, New York 14853, United States; ∥ Kavli Institute at Cornell for Nanoscale Science, Ithaca, New York 14853, United States; ⊥ Leibniz-Institut für Kristallzüchtung, Max-Born-Str. 2, Berlin 12489, Germany

**Keywords:** Interface, metastable, molecular beam epitaxy, thin film, functional materials, ferroelectric, multiferroic

## Abstract

The metastable *P*6_3_ *cm* phase of ScFeO_3_ (h-ScFeO_3_) is a multiferroic material, but instability on conductive substrates limits the development of next-generation memory and magnetoelectric sensors. Unfortunately, stabilization approaches developed for insulating substrates, such as sapphire, do not translate directly to conductive substrates. In this work, we demonstrate how interlayer design preferentially stabilizes h-ScFeO_3_ on (111) platinum via molecular beam epitaxy while simultaneously enhancing key figures of merit. We developed a process to deposit a (111) wüstite-like interlayer with a metastable Fe^3+^ oxidation state to target h-ScFeO_3_. The films are solely (0001) oriented h-ScFeO_3_ without any measured secondary phases. Rocking curves of the 0004 h-ScFeO_3_ peak have a full width at half-maximum of 0.06°, an improvement compared to films deposited without this interlayer approach. A further indication of strain reduction in these films is structural distortion in the first layers of h-ScFeO_3_, overcoming the critical thickness limit in h-ScFeO_3_. Designing interlayers to reduce epitaxial strain and target specific phases expands the viable substrates for metastable materials and overcomes the thickness limits for improper ferroelectricity.

## Graphical abstract

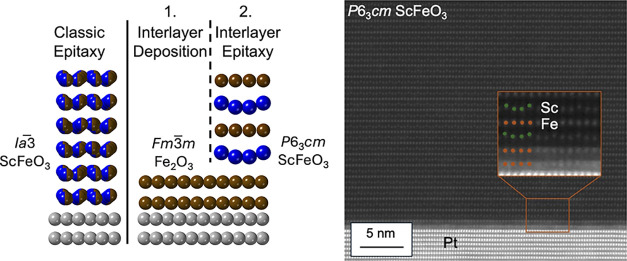

## Introduction

The *P*6_3_ *cm* phase of ScFeO_3_ (h-ScFeO_3_) is simultaneously ferroelectric and antiferromagnetic,^1,2^ making it ideal for many multiferroic applications, such as scalable magnetoelectric spin–orbit logic and magnetic biosensing.^3,4^ Thin films of h-ScFeO_3_ have the highest reported Néel transition temperature of rare-earth ferrites, up to 195 K,^1^ while maintaining improper ferroelectricity up to 681 K.^2,5^ Because the h-ScFeO_3_ phase is metastable and in competition with four other known polymorphs, routes to preferentially select and stabilize this phase are necessary.^6^ Stromataxic and interlayer stabilization have been developed to stabilize h-ScFeO_3_ and analogous structures,^5,7,8^ but these methods were developed for nonconductive substrates, such as Al_2_O_3_. Unfortunately, depositing h-ScFeO_3_ on the conductive substrates needed for multiferroic applications induces secondary phases or destabilizes this phase completely.^1^ Conductive substrates are needed for many multiferroic logic and sensors to extract charge or apply voltage to the material. While ScFeO_3_ deposited on oxide electrodes has enabled the study of the intrinsic properties of h-ScFeO_3_ (magnetic properties, ferroelectric switching),^1,2^ a critical next step to expand the applications of h-ScFeO_3_ is to stabilize the phase on device-compatible conductive substrates.

Scaling down the film thickness while maintaining polar distortion is critical to meet device scaling needs; however, interfacial clamping due to strain is an external limit to the critical thickness for polarization in improper ferroelectric materials like h-ScFeO_3_.^5,9,10^ Therefore, stabilizing h-ScFeO_3_ on a highly conductive substrate, such as (111) Pt, while simultaneously developing interlayers to decrease interfacial clamping is a critical step toward the application of h-ScFeO_3_ as an exemplary improper ferroelectric.^5,11^ Introducing an interlayer between a film and the substrate has long been used for interface strain mitigation,^12^ but further work is needed to understand the design criteria for using interlayers to select specific metastable phases. We have previously shown that h-ScFeO_3_ is stabilized on Al_2_O_3_ substrates by a wüstite iron oxide interlayer that forms spontaneously during deposition.^8^ The mechanisms of stabilization are proposed to be due to the similar oxygen sublattice between the wüstite iron oxide interlayer and the h-ScFeO_3_, the similar iron oxide layer structure, and the low calculated epitaxial strain (3.67% for h-ScFeO_3_ 112̅0 || Fe_*x*_O 1̅1̅2).^8^ Unfortunately, wüstite does not spontaneously form or precipitate out naturally on conductive substrates, potentially due to charge transfer and changes in lattice mismatch.^13^ Wüstite iron oxide would be compatible with deposition on several conductive materials, including (111) Pt, on which it can form in a metastable Fe^3+^ oxidation state.^13–16^ Therefore, it is necessary to intentionally design a chemically and structurally compatible interlayer deposition approach that can be translated to conductive substrates and reduce interfacial clamping.

This work investigates how to design an interlayer to stabilize the metastable *P*6_3_ *cm* phase of ScFeO_3_ on conductive substrates. We hypothesize that depositing a wüstite Fe_2_O_3_ interlayer on (111) Pt will preferentially stabilize the h-ScFeO_3_ phase because of the similar oxygen sublattice with h-ScFeO_3_ and flexible bond coordination in Fe_2_O_3_. To test this hypothesis, the phase formations of ScFeO_3_ films deposited both with and without a wüstite Fe_2_O_3_ interlayer are compared. We find that on (111) platinum, only films deposited with a wüstite bilayer form the *P*6_3_ *cm* phase of ScFeO_3_. We further developed a process to suppress the formation of secondary phases, resulting in highly crystalline h-ScFeO_3_. Furthermore, the overlap of the oxygen sublattices and the low epitaxial strain reduce the interfacial clamping compared to previous reports, paving the way to reducing the critical thickness of ferroelectric devices.

## Results and Discussion

Given that the *P*6_3_ *cm* phase of ScFeO_3_ (h-ScFeO_3_) is metastable, the first step is to determine the deposition conditions necessary to stabilize the phase. Since Al_2_O_3_ has previously been observed to stabilize h-ScFeO_3_,^6–8^ initial depositions focus on this substrate. The scandium and iron fluxes were alternated to promote the stabilization of the layered h-ScFeO_3_ phase using a procedure adapted from reference.^7^ Figure S1a shows the X-ray diffraction (XRD) of a ScFeO_3_ film deposited at 500 °C. At or below 500 °C, the peaks observed in XRD fit to the ground state bixbyite ScFeO_3_ phase. Increasing the temperature above 800 °C leads to the formation of the *P*6_3_ *cm* phase. The X-ray diffraction (XRD) of a representative h-ScFeO_3_ thin film deposited on Al_2_O_3_ at 900 °C is shown in Figure 1a. The diffraction pattern exhibiting 000 *2l* fits to the *P*6_3_ *cm* phase of ScFeO_3_ with a (0001) orientation.^17^ The rocking curve, taken from the 0004 h-ScFeO_3_ peak, in the inset of Figure 1a, demonstrates the degree of crystallographic alignment of the film. The full width at half-maximum (FWHM) of the rocking curve is 0.42°, on par with the lowest values currently reported in literature, 0.4°.^17^

**Figure 1:**
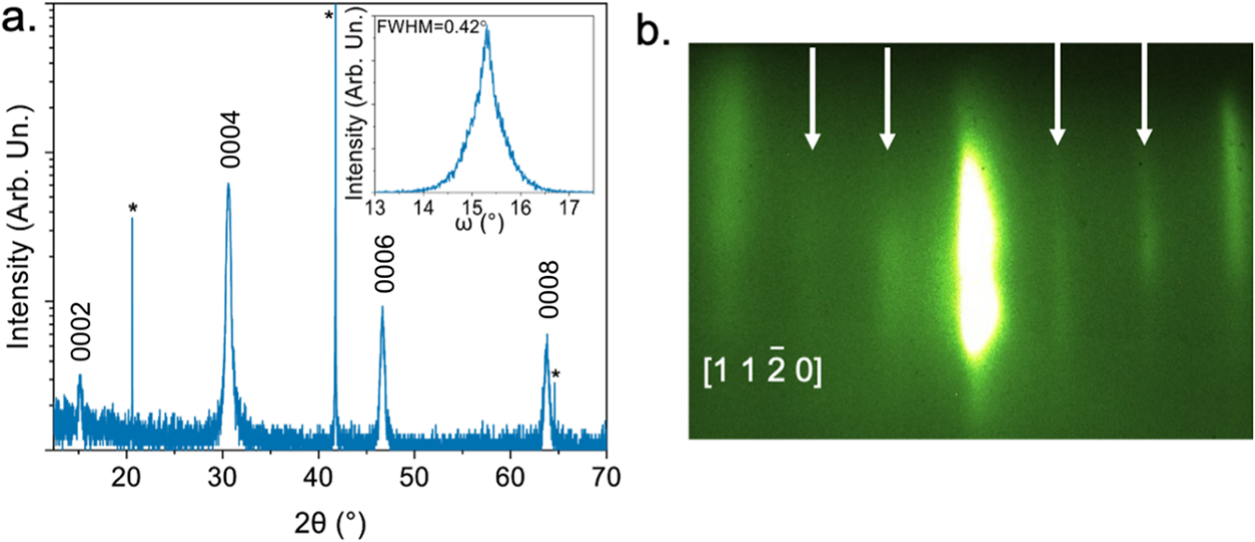
(a) XRD pattern of a h-ScFeO_3_ film deposited on (0001) Al_2_O_3_. Reflections marked with 000 *2l* are from the *P*6_3_ *cm* phase and reflections marked with * are from the substrate; inset rocking curve measurement of the 0004 h-ScFeO_3_ reflection; (b) RHEED taken along the h-ScFeO_3_ [1 1 2̅ 0] direction after cooling. Arrows indicate the reconstruction from the scandium displacement.

Reflection high-energy electron diffraction (RHEED) taken during deposition further corroborates the formation of the *P*6_3_ *cm* phase. Figure 1b shows the RHEED along the h-ScFeO_3_ [1 12̅ 0] direction from the same film in Figure 1a after 60 min of deposition and cooling to room temperature. The ±(3*n* + 1)/3 and ±(3*n* + 2)/3 diffraction streaks (highlighted with the white arrows) are clear indications of the √3 × √3 reconstruction in the *a*–*b* plane induced by the structural distortion of the scandium atoms associated with the *P*6_3_ *cm* phase.^5^ These distinctive reconstructions have previously been reported for many isostructural complex transition metal oxides.^5,18^ The RHEED also provides a clear means to differentiate between the metastable hexagonal phase and bixbyite ground state because the ground state has a clearly different in-plane diffraction pattern, like that seen for the predominantly bixbyite ScFeO_3_ films deposited at 500 °C in Figure S1b.

Another indication of the quality of the films is the low surface roughness of the h-ScFeO_3_ films deposited onto sapphire substrates. Figure S2 shows 5 x 5 μm atomic force microscopy (AFM) images taken from the surface of an h-ScFeO_3_ film. The average surface roughness (*R*_a_) of the films is approximately 0.5 nm, which is on par with previous reports of other high-quality oxide films deposited by MBE.^19,20^

Unfortunately, the same conditions that stabilize the *P*6_3_ *cm* phase of ScFeO_3_ on Al_2_O_3_ result in the ground state bixbyite phase when the films are deposited onto a metallic substrate, such as (111) Pt.^1^ Figure S3a,b show the XRD and RHEED patterns for these ScFeO_3_ films deposited under the same conditions as those used for the sample shown in Figure 1a. On platinum substrates, the films form in the bixbyite phase under the same conditions that resulted in stabilization of the *P*6_3_ *cm* phase on Al_2_O_3_. The platinum films have different emissivity and thermal conductivity than sapphire, which suggests that a different range of deposition temperatures could be used to reach the same phase. Nonetheless, adjusting the temperature from 700 to 1100 °C still results in only the ground state bixbyite phase, as seen by the RHEED in Figure S3c,d. The inability to stabilize h-ScFeO_3_ on platinum is unexpected, given the closer one-dimensional lattice mismatch of −3.4% compared to −17.1% on Al_2_O_3_. However, our prior work has shown that when ScFeO_3_ is deposited onto Al_2_O_3_, an interlayer of (111)-oriented wüstite Fe_*x*_O bilayer forms that then stabilizes h-ScFeO_3_ by providing a matching iron–oxygen sublattice.^8^ These results suggest that the stabilization mechanism that occurs on Al_2_O_3_ does not intrinsically occur on platinum.^8,21^ Thus, we propose to intentionally design an iron oxide interlayer that will preferentially stabilize h-ScFeO_3_ on conductive substrates. While the magnitude of the one-dimensional lattice mismatch of h-ScFeO_3_ on (111) wüstite (3.6%) is comparable to that of h-ScFeO_3_ on (111) Pt (−3.4%), adding a wüstite iron oxide interlayer is predicted to preferentially select for the h-ScFeO_3_ phase because of the coordination and bonding flexibility of the iron.^21^ Additionally, the similarity between the oxygen sublattice between the interlayer and h-ScFeO_3_ has been observed to preferentially stabilize the desired metastable phase.

Depositing an interlayer should stabilize the phase; however, the (111) wüstite FeO that forms on Al_2_O_3_ does not form under the same conditions as on platinum. While the wüstite phase of FeO is stable on Al_2_O_3_ at substrate temperatures above 250 °C,^22,23^ the reported process for stabilization is different on (111) Pt.^14,15^ Furthermore, the oxidation state of the wüstite phase can range from a stable Fe^2+^ oxidation state to a metastable Fe^3+^ oxidation state depending on the oxidizing species used. To deposit Fe_*x*_O on (111) Pt films, a two-step process is used—metallic iron is first deposited at room temperature and then subsequently oxidized at temperatures above 600 °C.^8,14–16,23^ We use a similar approach here to target the deposition of a two-atomic-layer-thick, (111)-oriented, wüstite iron oxide interlayer by adapting the methods from Ritter et al.^14^ A differentiating factor in this work is that an 80% ozone source is used to target the Fe^3+^ oxidation state to match the oxidation state of the interlayer and h-ScFeO_3_, aiming to reduce the mismatch between the interlayer and film. The formation of the wüstite iron oxide interlayer is evident in the RHEED images in Figure S4a,b. The RHEED patterns, taken after oxidation along the Fe_2_O_3_ [1 1̅ 0] and [112̅], match those previously reported for wüstite Fe_2_O_3_.^23^ AFM images of the platinum substrate (Figure S4c) and the iron oxide interlayer (Figure S4d) both have an *R*_a_ = 0.3 nm, confirming that the low surface roughness of the substrate is maintained following interlayer deposition. Now that we have achieved the desired (111)-oriented wüstite iron oxide interlayer, the next step is to determine if this phase will provide a platform for the stabilization of h-ScFeO_3_.

From the XRD, we see that our hypothesis is correct that an intentional addition of a (111)-oriented wüstite iron oxide interlayer stabilizes the h-ScFeO_3_ phase. Figure 2 highlights the importance of depositing the Fe_2_O_3_ interlayer to stabilize the *P*6_3_ *cm* phase of ScFeO_3_. Starting the deposition onto Pt with a single layer of scandium oxide or iron oxide at 900 °C (deposition temperature) results in solely (111)-oriented bixbyite ScFeO_3_. Only after adding a (111) wüstite interlayer does the (0001)-oriented h-ScFeO_3_ form. For films deposited onto an interlayer, no other secondary phases of ScFeO_3_ are observed. However, there are peaks consistent with (111)-oriented *Fd*3̅*m* Fe_3_O_4_ (PDF #01–080–6410).^24^ The *c*-axis lattice parameter of the h-ScFeO_3_ films deposited on the interlayer is calculated to be 11.7 Å, which matches the lattice parameter calculated for the film deposited on Al_2_O_3_. Stabilization is likely not solely due to epitaxial strain, as the −3.8% lattice mismatch between h-ScFeO_3_ and the interlayer is larger in magnitude than the 3.2% lattice mismatch between h-ScFeO_3_ and platinum. Therefore, the overlap between the oxygen sublattice of h-ScFeO_3_ and the wüstite interlayer and the similarity in the iron sublattices also play a role in stabilization.^8^ By developing this interfacial layer between ScFeO_3_ and platinum, we stabilized h-ScFeO_3_, which is a critical step to expand the applications of this phase.

**Figure 2:**
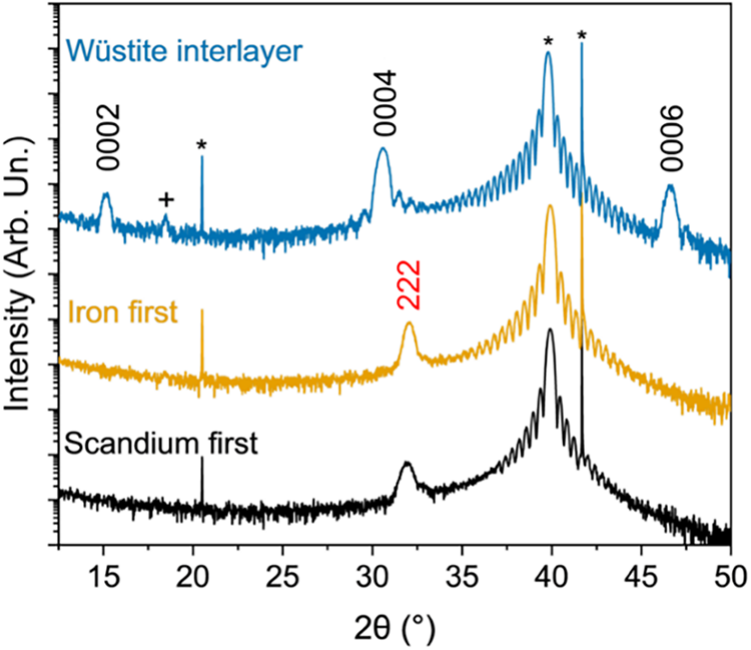
XRD patterns of ScFeO_3_ films on (111) Pt-coated (0001) Al_2_O_3_ substrates deposited with or without an intentional iron oxide interlayer. The 000 *2l* reflections fit to h-ScFeO_3_, the *hkl* reflections are fit to bixbyite ScFeO_3_, the + reflections are fit to (111) Fe_3_O_4_, and the * reflections are fit to (111) Pt and the Al_2_O_3_ substrate.

While using an interlayer that includes one or more elements from the desired phase is advantageous, as it can template growth due to similar sublattices, it can also lead to nonstoichiometry in subsequent films. In the films deposited with an Fe_2_O_3_ interlayer using the same conditions used for deposition on Al_2_O_3_, a peak corresponding to (111)-oriented *Fd*3̅*m* Fe_3_O_4_ (magnetite) is seen by XRD in Figure 2. On (111) Pt, the wüstite Fe_2_O_3_ phase is metastable and transitions to magnetite at thicknesses above 2–3 layers.^14,23^ These reports are consistent with finding magnetite as a secondary phase rather than other iron oxide phases. As previously reported, magnetite precipitation occurs in these h-ScFeO_3_ films deposited on the interlayer. Figure S5a gives an example of an AFM micrograph with faceted islands that occurred in and on thin films processed with an excess of iron. Energy dispersive X-ray spectroscopy (EDS) results of the scanning electron micrograph in Figure S5b corroborate that the faceted islands are iron-rich compared to the rest of the h-ScFeO_3_ films. Similar faceted islands have previously been attributed to iron excess in isostructural LuFeO_3_.^25^ Furthermore, in the RHEED pattern in Figure S5c, there are dim diffraction streaks that fit to the Fe_3_O_4_. The critical issue is not just the formation of a secondary phase but its impact on the surface roughness and conductivity through the thickness of the film. The conductivity of Fe_3_O_4_ is 2 × 10^2^ Ω^–1^ cm^–1^ at room temperature, and therefore would be detrimental in an electronic or optoelectronic device.^26^ Due to the formation of the secondary Fe_3_O_4_ phase, it is critical to find a way to reduce the excess iron in these films.

To reduce the excess iron in the ScFeO_3_ films with an iron oxide interlayer, the time of iron deposition is reduced by 10% in the first eight deposition cycles after depositing the iron oxide bilayer. This process leads to the disappearance of the iron oxide peaks in the 2θ scans, as seen in the XRD data for an h-ScFeO_3_ film deposited on a wüstite interlayer on (111) Pt at 900 °C in Figure 3a. Furthermore, the 000 *2l* peaks of h-ScFeO_3_ have Laue oscillations, an indication that the films are homogeneous and have smooth interfaces.^27^ Rocking curves of the h-ScFeO_3_ film deposited with the reduced iron flux on the Fe_2_O_3_ interface layer provide further insight into the degree of crystalline orientation. The FWHM of the rocking curve is 0.06°, as seen in the inset of Figure 3a, which is the lowest value reported for h-ScFeO_3_ in the literature. Compared to the rocking curves of films with excess iron in Figure S6, this is a 2-fold decrease in rocking curve FWHM. Beyond crystalline orientation, rocking curves of isostructural materials have been used to determine if films are stoichiometric.^25^ In a previous study, it was found that in structurally analogous LuFeO_3_, an excess of either cation resulted in a broadening of the rocking, with the FWHM ranging from 0.6 to 1.6°.^25^ Therefore, the narrow rocking curve of the h-ScFeO_3_ films deposited on platinum with an iron oxide interlayer is an additional indication that these films are near stoichiometry. With the improved rocking curve, there is also a significant reduction in the size and concentration of faceted islands at the film surface, as shown in Figure 3b. The AFM further supports the reduction in the excess of iron. The R_a_ decreases from 1.5 to 0.6 nm when depositing with a deficiency of iron in the first layers. Furthermore, in Figure 3c, the Fe_3_O_4_ diffraction pattern is not visible in the RHEED of an exemplary film, showing that the formation of surface iron oxides is suppressed. Overall, depositing with a 10% reduction of iron in the first eight cycles not only reduces iron oxide formation but also enhances the crystallinity of the h-ScFeO_3_.

**Figure 3:**
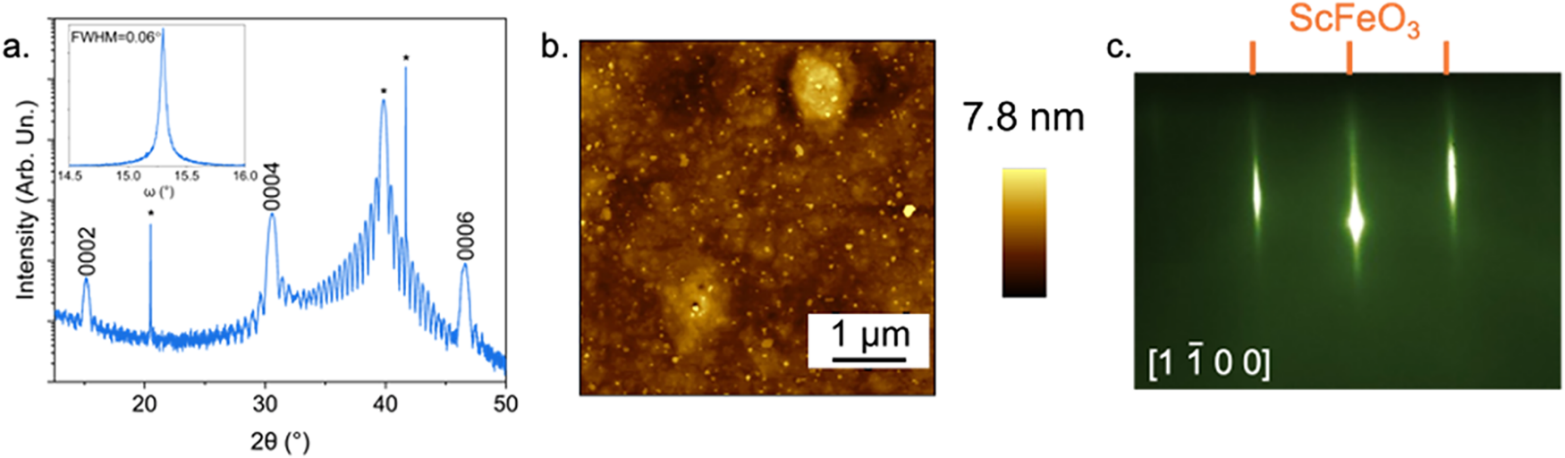
(a). XRD of a representative h-ScFeO_3_ film deposited on a wüstite interlayer with reduced iron flux in the first eight layers; inset rocking curve measurement of the 0004 h-ScFeO_3_ reflection. (b) AFM image of the film shown in panel (a). (c) RHEED of the film at the deposition temperature of 900 °C.

Scanning transmission electron microscopy (STEM) provides further evidence for the interlayer stabilization of crystalline h-ScFeO_3_ on (111) Pt, as seen in Figure 4a. The layered structure of scandium and iron in the h-ScFeO_3_ phase is clearly visible by STEM. The down–down–up displacement of the scandium atoms is also visible in the image, which is the key feature that defines the structural distortion along the 6-fold rotational axis in the *P*6_3_ *cm* phase of ScFeO_3_. H-ScFeO_3_ is known to have two distinct polarization states, arising from up–up-down polar and down–down–up polar distortions of the scandium lattice. Both polar distortions are observed via STEM (Figure S7), indicating that the two polarization directions have comparable energies, as expected for a ferroelectric material. A similar domain wall between the two polarization directions was observed in h-ScFeO_3_ deposited on the La_0.8_Sr_0.2_MnO_3_ substrates. Microscopic polarization switching was assessed using piezoresponse force microscopy (PFM) following a poling procedure described in ref 28. Two stable polarization states were measured 30 min after poling, from the +9 and −9 V biases applied to different regions of an exemplary film, as shown in Figure S8. Although some backswitching was observed, the majority of the poled regions retain polarization. The observed switchable polarization of h-ScFeO_3_ observed by PFM is consistent with previous measurements of ferroelectricity in this material and other isostructural materials.^1,29,30^

**Figure 4:**
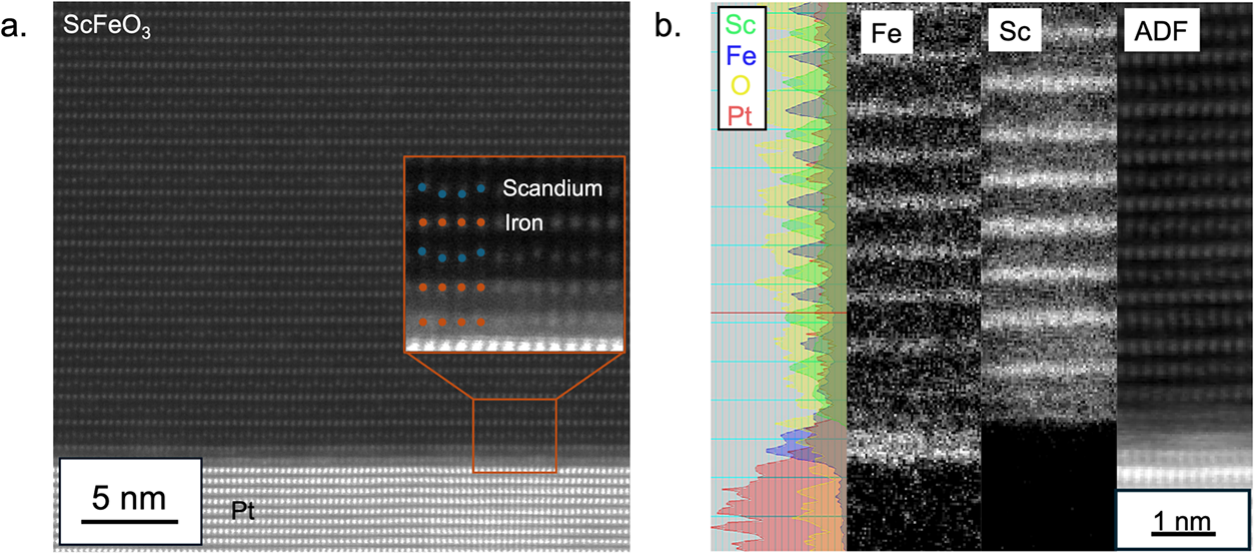
(a) Scanning transmission electron microscope image of the h-ScFeO_3_ interface, iron oxide interlayer, and platinum films taken along the [11 2̅0] h-ScFeO_3_ zone axis. Inset is a zoomed-in region of the film at the film–substrate interface with a schematic of the atomic positions overlaid. (b) Electron energy loss spectroscopy of an h-ScFeO_3_ film on (111) Pt deposited with an iron oxide interlayer.

In prior studies, a common limitation in rare-earth hexagonal ferrites and manganites is that the structural distortion is not present in the first couple of layers due to interfacial clamping from the substrate.^5,9,11^ In the films deposited on the iron oxide interlayer, structural distortion is visible in the first layer of scandium in the film. The structural distortion throughout the film is a further indication of the importance of interlayer design for scaling down the thickness of h-ScFeO_3_.^11^ Furthermore, the STEM images provide evidence of an intentionally deposited iron oxide bilayer. In Figure 4b, the iron and scandium composition of the atomic layers is determined using electron energy loss spectroscopy, showing the layered structure of h-ScFeO_3_. Analysis of the Fe-L_2,3_ edge (Figure S9) shows no change in the onset energy of the L_3_ edge between the interlayer and the h-ScFeO_3_ film, and the fine structure in both regions is consistent with the Fe^3+^ oxidation state.^31^ While the wüstite phase has an Fe^2+^ oxidation state in bulk, a wüstite phase with a metastable Fe^3+^ oxidation state has been observed on platinum.^21,23^ The oxidation state of the intentionally deposited interlayer matches the oxidation state in h-ScFeO_3_, which likely causes the high crystalline quality in the films in this work. This work demonstrates that interlayers are critical in expanding the deposition of hexagonal ferrites to other substrates due to a lack of commercial substrates with an epitaxial match.

Collectively, the XRD, STEM, and RHEED results all indicate that a wüstite interlayer is critical to the stabilization of metastable h-ScFeO_3_. The stabilization of the phase was observed only when this interlayer was deposited, and the structural distortion in the first layer is an indication of the reduced interfacial clamping. All films deposited using the interlayer stabilization method in this study resulted in h-ScFeO_3_ phase stabilization for 17 subsequent depositions when depositing ScFeO_3_ between 850 and 900 °C, demonstrating the reproducibility of this method. For conductive substrates such as platinum, the interlayer must be specifically designed and intentionally added, whereas on substrates such as sapphire, an interlayer forms spontaneously. The difference in stabilization approach on platinum compared to Al_2_O_3_ is because FeO is stable on (0001) Al_2_O_3_ when depositing at temperatures above 250 °C and with an oxygen atmosphere,^22,23^ while on (111) Pt an iron layer must be initially deposited at room temperature and subsequently oxidized.^14,15^ While the thickness and crystal structure match interlayers observed in thin films of h-ScFeO_3_ and h-LuFeO_3_ formed during pulsed laser deposition, the oxidation state of the interlayer phase does not match, either due to different interactions with the substrate or the change in deposition conditions.^8,21^ The STEM images show that the structural distortion of the scandium atoms is present in the first layer (and subsequent scandium layers), which was not previously observed by RHEED of h-ScFeO_3_ deposited by pulsed laser deposition.^5^ The interlayer deposition procedure should be tested on thinner films (i.e., only a couple of unit cells) to test whether the structural distortion is present at or near the monolayer limit, as has been observed in LuFeO_3_ films deposited on SrCo_2_Ru_4_O_11_ substrates.^11^ The Fe_2_O_3_ phase used to stabilize h-ScFeO_3_ is also stable on (001) Cu, (0001) Ru, and other conductive substrates, expanding the viable bottom electrodes and substrate materials for the stabilization of rare-earth hexagonal ferrites.^32^ While deposition on oxide substrates has been critical to understand the fundamental properties of ScFeO_3_, the expanded substrates available for phase stabilization from this process will enable multiferroic memory and magnetoelectric sensing. The structural compatibility of h-ScFeO_3_ with the iron oxide bilayers of varied oxidation states indicates that a similar superlattice could be formed to that of LuFeO_3_/LuFe_2_O_4_, a room temperature multiferroic.^8,33^ There also exists an isostructural *Fm*3̅*m* phase of MnO with similar lattice parameters compared to FeO.^34^ Therefore, the same designed interlayer approach could be applied to rare-earth manganites using MnO as an interlayer. The method to stabilize h-ScFeO_3_ described in this work provides a pathway to stabilizing other hexagonal rare-earth materials and can be applied to a wide range of substrate materials with an interlayer approach.

## Conclusions

The development of a wüstite Fe_2_O_3_ interlayer enables the stabilization of the metastable *P*6_3_ *cm* phase of ScFeO_3_ on conductive (111) Pt. Developing the interlayer is critical, as the optimal conditions found for film deposition on Al_2_O_3_ led to films with the ground state bixbyite structure upon depositing directly on (111) Pt. The interlayer enables the stabilization of 0001-oriented *P*6_3_ *cm* ScFeO_3_, improving the rocking curve FWHM from 0.4 to 0.06°. While Fe_3_O_4_ precipitates can form at the surface, these were reduced by depositing a deficiency of iron in the subsequent layers. The crystallinity of the h-ScFeO_3_ thin films is further confirmed by STEM, where structural distortion of the scandium lattice is present through the thickness of the film. The oxidation state of the iron interlayer is found to be Fe^3+^, in contrast to the expected oxidation state of wüstite (Fe^2+^). Overall, the method developed here is an ideal interlayer to stabilize h-ScFeO_3_ and other hexagonal ferrites on conductive substrates to explore the magnetoelectric applications of h-ScFeO_3_.

## Methods

ScFeO_3_ films were deposited by molecular beam epitaxy (MBE) (Veeco Gen10) at the NSF PARADIM MBE Facility at Cornell University. Initial experiments focused on the deposition of h-ScFeO_3_ on Al_2_O_3_ because this substrate has previously been shown to stabilize h-ScFeO_3_, despite having a lattice mismatch of −17.1%.^8^ The single-crystal sapphire (0001) Al_2_O_3_ substrates (CrysTec, GmbH) were annealed in the MBE chamber at 1500 °C to remove surface contamination and improve surface smoothness. The substrates were then cooled to the deposition temperature and subsequently allowed to equilibrate at that temperature for 20 min prior to thin film deposition. For thin film growth, the substrates were heated to temperatures ranging from 500 to 1100 °C, using Epiray’s THERMALAS heater featuring a Coherent 10.6 μm CO_2_ laser. The substrate temperature was monitored by using a Heitronics optical pyrometer operating at 7.5 μm.

Elemental scandium and iron were loaded into tungsten and alumina crucibles, respectively, and supplied from independent medium temperature effusion cells. A quartz crystal microbalance (QCM) was used to provide preliminary flux calibrations. Since QCMs often have an error above 10%, a binary oxide calibration was also used to determine the flux rates within an accuracy of <1% error for the iron and scandium, using a process described in reference 35. The binary oxide calibration was performed by depositing films of Sc_2_O_3_ and Fe_3_O_4_ and using the film thickness of each oxide, determined by X-ray reflectivity (XRR), to calibrate the elemental flux rate. XRR was conducted on a Malvern Panalytical Empyrean with a copper Kα source (λ = 1.5406) with a four-bounce Ge (220) monochromator, with a step size of 0.02° with a speed of 4° min^–1^. The thickness was then calculated using the spacing of the Kiessig fringes using the Smartlab Studio II software (Rigaku). An equal flux rate of iron and scandium of 2.5 × 10^13^ atoms cm^–2^ s^–1^ was targeted for all depositions, and the effusion cell temperature was adjusted until the flux was measured below 2% deviation.

Figure S10a,b shows the XRR data taken for thin films of (111) *Ia*3̅ Sc_2_O_3_ and (001) *R*3̅*c* Fe_2_O_3_, respectively. The thickness and density for each film are listed in Table S1 that were used to calculate the flux rate and deposition timing for each sample. The flux of iron is calculated to be 2.53 × 10^13^ atoms cm^–2^ s^–1^ and the flux of scandium is calculated to be 2.48 × 10^13^ atoms cm^–2^ s^–1^. Layer timing was then calculated using the planar atomic density of h-ScFeO_3_, which is 1.07 × 10^15^ atoms cm^–2^ for both scandium and iron.^17^

Once the individual flux rates were determined, the ScFeO_3_ film deposition occurred in discrete layers of scandium and iron, following a methodology similar to that described in reference 7. The layer timing for ScFeO_3_ film deposition was set to alternate with the deposition of scandium and iron to target the layered structure of the *P*6_3_ *cm* phase of ScFeO_3_. The shutter timing was adjusted to account for any variations in the measured flux rate compared to the targeted rate (2.5 × 10^13^ atoms cm^–2^ s^–1^) to keep the number of atoms deposited per layer consistent. The base pressure of the chamber was below 2 × 10^–7^ Torr prior to all depositions. An oxidant partial pressure of 1 × 10^–6^ Torr with 80% O_3_ was used for all depositions of ScFeO_3_. The ScFeO_3_ film phase formation was monitored by in situ reflection high-energy electron diffraction (RHEED) (STAIB Instruments) operating at 14 kV and 1.4–1.5 A during film growth and subsequent cooling.

For the deposition onto conductive substrates, a layer of platinum was deposited on annealed (0001) Al_2_O_3_ single-crystal substrates (Sapphire, CrysTec, GmbH) by electron beam deposition prior to the ScFeO_3_ film deposition without breaking the vacuum between depositions. Platinum was chosen because (111) platinum offers a conductive substrate and because it can be used to target the desired interlayer of (111) wüstite. Further information on the deposition conditions for the platinum layer can be found in reference 36. The resulting (111) platinum films exhibit a narrow rocking curve full width at half-maximum of 0.004°, a surface roughness of 0.20 nm, and conductivity of 8.99 × 10^6^ S/m.

A wüstite iron oxide interlayer is used to stabilize h-ScFeO_3_ due to the overlap in the oxygen sublattice and low epitaxial strain (−3.7%). The deposition process used to target the wüstite iron oxide interlayer was developed based on reference 14. Iron was initially deposited at room temperature by MBE onto (111) Pt substrates, and then, the films were heated to 600 °C in an oxidant atmosphere of 1 × 10^–7^ Torr of 80% O_3_ to target an Fe^3+^ oxidation state. After the oxidation step, the temperature of the substrates was increased to 900 °C for the deposition of h-ScFeO_3_ by using alternating scandium and iron fluxes for the rest of the deposition.

The morphology of the films was determined from atomic force microscopy (AFM) using an Asylum Research Cypher ES with Arrow UHF tips (NanoWorld, < 10 nm radius, silicon with reflective aluminum coating). A tapping AFM mode was used at a scan rate of 5 Hz and 512 samples/line. Piezoresponse force microscopy (PFM) images were collected using a Bruker Icon AFM with SCM-PIT (Bruker, <25 nm radius, antimony-doped silicon with 20 nm PtIr coating) probes. DC fields of +9 and −9 V were applied to pole the sample with a scan rate of 0.996 Hz and 256 samples/line. The bottom electrode was grounded by applying silver paint to the side of the sample. To measure the phase and amplitude from the poled sample, an AC of 2 V was applied to the sample with a single frequency below contact resonance, a scan rate of 0.5 Hz, and 512 samples/line. Scanning electron microscopy (SEM) and energy dispersive X-ray spectroscopy (EDS) measurements were taken on the ScFeO_3_ films using a Thermo Axia ChemiSEM. The voltages for SEM and EDS were 20 kV. SEM images were taken with an Everhart-Thornley secondary electron detector. EDS was collected with a TrueSight X EDS detector with a takeoff angle of 35°.

A cross-section of the interface for scanning transmission electron microscopy (STEM) was prepared by using a Thermo Fisher Scientific Helios G4UX focused ion beam. High-angle annular darkfield STEM images were acquired using a Thermo Fisher Scientific Spectra 300 equipped with a cold field emission source operated at an accelerating voltage of 300 kV, a semiconvergence angle of 30 mrad, and a probe current of 60 pA. Electron energy loss spectroscopy was acquired using a Gatan Continuum spectrometer.

## Supplementary Material



## Notes

XRD and RHEED of bixbyite ScFeO_3_, AFM of h-ScFeO_3_ on Al_2_O_3_, XRD and RHEED of bixbyite ScFeO_3_ deposited on Pt, RHEED of the Fe_2_O_3_ interlayer, AFM, SEM, RHEED, and rocking curve of h-ScFeO_3_ with excess iron, PFM of h-ScFeO_3_, Fe-L_2,3_ edge EELS spectra, and XRR data and fit parameters used to adjust the deposition timing (PDF)

## References

[1] Hamasaki Y., Katayama T., Yasui S., Shiraishi T., Akama A., Kiguchi T., Taniyama T., Itoh M. (2020). Switchable
Third ScFeO_3_ Polar Ferromagnet with YMnO_3_-Type
Structure. J. Mater. Chem. C.

[2] Sinha K., Wang H., Wang X., Zhou L., Yin Y., Wang W., Cheng X., Keavney D. J., Cao H., Liu Y., Wu X., Xu X. (2018). Tuning the Néel Temperature
of Hexagonal Ferrites by Structural Distortion. Phys. Rev. Lett..

[3] Sahini, M. G. ; Malima, N. M. Chapter 14 - Multiferroic Magnetoelectric-Based Biosensors in Healthcare. In Fundamentals of Biosensors in Healthcare; Hasnain, M. S. ; Nayak, A. K. ; Aminabhavi, T. M. , Eds.; Academic Press, 2025; pp 337–357 10.1016/B978-0-443-21658-9.00025-5.

[4] Manipatruni S., Nikonov D. E., Lin C.-C., Gosavi T. A., Liu H., Prasad B., Huang Y.-L., Bonturim E., Ramesh R., Young I. A. (2019). Scalable Energy-Efficient Magnetoelectric Spin–Orbit
Logic. Nature.

[5] Li X., Yun Y., Xu X. (2023). Improper Ferroelectricity
in Ultrathin Hexagonal Ferrites
Films. Appl. Phys. Lett..

[6] Hamasaki Y., Shimizu T., Yasui S., Taniyama T., Sakata O., Itoh M. (2016). Crystal Isomers of
ScFeO_3_. Cryst.
Growth Des..

[7] Garten L. M., Jiang Z., Paik H., Perkins J. D., Kakekhani A., Fei R., Werder D. J., Holtz M. E., Ginley D. S., Rappe A. M., Schlom D. G., Staruch M. L. (2021). Stromataxic Stabilization of a Metastable
Layered ScFeO_3_ Polymorph. Chem. Mater..

[8] Frye M. B., Tian M., Moynihan E., Sanchez A., Garten L. M. (2025). Interlayer-Mediated
Stabilization of Metastable P6_3_cm ScFeO_3_ on
Al_2_O_3_. Adv. Mater. Interfaces.

[9] Norlander J., Campanini M., Rossell M. D., Erni R., Meier Q. N., Cano A., Spaldin N. A., Fiebig M., Trassin M. (2019). The Ultrathin
Limit of Improper Ferroelectricity. Nat. Commun..

[10] Li X., Yun Y., Xu X. (2025). Recent Progress on Multiferroic Hexagonal Rare-Earth
Ferrites (h-RFeO_3_, R = Y, Dy-Lu). J. Phys. Appl. Phys..

[11] Li, Y. E. ; KP, H. ; Lu, H. ; Steinhardt, R. A. ; Holtz, M. E. ; Brützam, M. ; Dykes, M. M. ; Arenholz, E. ; Hazra, S. ; LaVopa, A. ; Huang, X. ; Zhao, W. ; Behera, P. ; Ramesh, M. ; Krysko, E. ; Gopalan, V. ; Ramesh, R. ; Fennie, C. J. ; Cava, R. J. ; Guguschev, C. ; Gruverman, A. ; Muller, D. A. ; Schlom, D. G. Improper Ferroelectricity at the Monolayer Limit, arXiv:2503.06214. arXiv.org e-Print archive. https://arxiv.org/abs/2503.06214. 2025. 10.1126/sciadv.aec5221 PMC13344360 42418585

[12] Liu B., Chen Q., Chen Z., Yang S., Shan J., Liu Z., Yin Y., Ren F., Zhang S., Wang R., Wu M., Hou R., Wei T., Wang J., Sun J., Li J., Liu Z., Liu Z., Gao P. (2022). Atomic Mechanism of
Strain Alleviation and Dislocation Reduction in Highly Mismatched
Remote Heteroepitaxy Using a Graphene Interlayer. Nano Lett..

[13] Giordano L., Lewandowski M., Groot I. M. N., Sun Y.-N., Goniakowski J., Noguera C., Shaikhutdinov S., Pacchioni G., Freund H.-J. (2010). Oxygen-Induced Transformations of
an FeO(111) Film
on Pt(111): A Combined DFT and STM Study. J.
Phys. Chem. C.

[14] Ritter M., Ranke W., Weiss W. (1998). Growth and
Structure of Ultrathin
FeO Films on Pt(111) Studied by STM and LEED. Phys. Rev. B.

[15] Vurens G. H., Maurice V., Salmeron M., Somorjai G. A. (1992). Growth,
Structure
and Chemical Properties of FeO Overlayers on Pt(100) and Pt(111). Surf. Sci..

[16] Weiss W., Ritter M. (1999). Metal Oxide Heteroepitaxy: Stranski-Krastanov
Growth
for Iron Oxides on Pt(111). Phys. Rev. B.

[17] Sinha, K. K. Growth and Characterization of Hexagonal Rare-Earth Ferrites (h-RFeO_3_; R = Sc, Lu, Yb). Ph.D., The University of NebraskaLincoln, United States -- Nebraska, https://www.proquest.com/docview/2036843691/abstract/70AC15D143F34470PQ/1 (accessed Nov 16, 2021).

[18] Wang W., Zhao J., Wang W., Gai Z., Balke N., Chi M., Lee H. N., Tian W., Zhu L., Cheng X., Keavney D. J., Yi J., Ward T. Z., Snijders P. C., Christen H. M., Wu W., Shen J., Xu X. (2013). Room-Temperature
Multiferroic Hexagonal LuFeO_3_ Films. Phys. Rev. Lett..

[19] Shi Q., Parsonnet E., Cheng X., Fedorova N., Peng R.-C., Fernandez A., Qualls A., Huang X., Chang X., Zhang H., Pesquera D., Das S., Nikonov D., Young I., Chen L.-Q., Martin L. W., Huang Y.-L., Íñiguez J., Ramesh R. (2022). The Role of Lattice
Dynamics in Ferroelectric Switching. Nat. Commun..

[20] Bai J., Yang J., Dong W., Zhang Y., Bai W., Tang X. (2017). Structural
and Magnetic Properties of Perovskite SrMnO_3_ Thin Films
Grown by Molecular Beam Epitaxy. Thin Solid
Films.

[21] Zhang Y., Si W., Yu R., Zhu J. (2021). Polyhedron and Charge Ordering in
Interfacial Reconstruction of a Hexagonal Ferrite/Sapphire Heterostructure. ACS Appl. Mater. Interfaces.

[22] Gota S., Guiot E., Henriot M., Gautier-Soyer M. (2000). Structural
Properties of Epitaxial Nanometric Iron Oxide Layers on α-Al_2_O_3_(0001): An in Situ RHEED Study during Growth. Surf. Sci..

[23] Gota S., Guiot E., Henriot M., Gautier-Soyer M. (1999). Atomic-Oxygen-Assisted
MBE Growth of Fe_2_O_3_: Metastable FeO(111)-like
Phase at Subnanometer Thicknesses. Phys. Rev.
B.

[24] Levy D., Giustetto R., Hoser A. (2012). Structure of Magnetite
(Fe_3_O_4_) above the Curie Temperature: A Cation
Ordering Study. Phys. Chem. Miner..

[25] Moyer J. A., Misra R., Mundy J. A., Brooks C. M., Heron J. T., Muller D. A., Schlom D. G., Schiffer P. (2014). Intrinsic Magnetic
Properties of Hexagonal LuFeO_3_ and the Effects of Nonstoichiometry. APL Mater..

[26] AC Properties of Ferrites. In Modern Ferrite Technology; Goldman, A. , Ed.; Springer US: Boston, MA, 2006; pp 35–50 10.1007/978-0-387-29413-1_3.

[27] Miller A. M., Lemon M., Choffel M. A., Rich S. R., Harvel F., Johnson D. C. (2022). Extracting Information from X-Ray
Diffraction Patterns
Containing Laue Oscillations. Z. Naturforsch..

[28] Pal S., Palladino E., Yuan H., de h-Óra M. A., MacManus-Driscoll J. L., Ontaneda J., Dwij V., Sathe V. G., Briscoe J. (2024). Determination of Imprint Effects in Ferroelectrics
from the Quantified Phase and Amplitude Response. ACS Appl. Electron. Mater..

[29] Du K., Gao B., Wang Y., Xu X., Kim J., Hu R., Huang F. T., Cheong S. W. (2018). Vortex
Ferroelectric Domains, Large-Loop
Weak Ferromagnetic Domains, and Their Decoupling in Hexagonal (Lu,
Sc)­FeO3. npj Quantum Mater..

[30] Casamento J., Holtz M. E., Paik H., Dang P., Steinhardt R., Xing H. Grace., Schlom D. G., Jena D. (2020). Multiferroic LuFeO_3_ on GaN by Molecular-Beam Epitaxy. Appl.
Phys. Lett..

[31] Tan H., Verbeeck J., Abakumov A., Van Tendeloo G. (2012). Oxidation
State and Chemical Shift Investigation in Transition Metal Oxides
by EELS. Ultramicroscopy.

[32] Palacio I., Monti M., Marco J. F., McCarty K. F., de la
Figuera J. (2013). Initial Stages of FeO Growth on Ru(0001). J. Phys.: Condens. Matter.

[33] Mundy J. A., Brooks C. M., Holtz M. E., Moyer J. A., Das H., Rébola A. F., Heron J. T., Clarkson J. D., Disseler S. M., Liu Z., Farhan A., Held R., Hovden R., Padgett E., Mao Q., Paik H., Misra R., Kourkoutis L. F., Arenholz E., Scholl A., Borchers J. A., Ratcliff W. D., Ramesh R., Fennie C. J., Schiffer P., Muller D. A., Schlom D. G. (2016). Atomically Engineered Ferroic Layers Yield a Room-Temperature
Magnetoelectric Multiferroic. Nature.

[34] Rizzi G. A., Petukhov M., Sambi M., Zanoni R., Perriello L., Granozzi G. (2001). An X-Ray Photoelectron Diffraction Structural Characterization
of an Epitaxial MnO Ultrathin Film on Pt(1 1 1). Surf. Sci..

[35] Sun J., Parzyck C. T., Lee J. H., Brooks C. M., Kourkoutis L. F., Ke X., Misra R., Schubert J., Hensling F. V., Barone M. R., Wang Z., Holtz M. E., Schreiber N. J., Song Q., Paik H., Heeg T., Muller D. A., Shen K. M., Schlom D. G. (2022). Canonical Approach to Cation Flux
Calibration in Oxide Molecular-Beam Epitaxy. Phys. Rev. Mater..

[36] Frye M. B., Chin J. R., Barone M., Zeltmann S. E., Garten L. M. (2025). Enhancing
the Conductivity and Crystallinity of (111) Platinum Films via a Two-Temperature
Deposition and Substrate Annealing. Scr. Mater..

