# Supplementary material for: Stabilizing Metastable Rare-Earth Ferrites on (111) Platinum via an Iron Oxide Interlayer
Source: ACS Appl Electron Mater. 2026 Mar 12;8(6):2420–7. doi: 10.1021/acsaelm.5c02616 (PMC13019661; doi:10.1021/acsaelm.5c02616)
Supplement: Supplementary file 1 [file el5c02616_si_001.pdf]

Supporting Information

Stabilizing Metastable Rare-Earth Ferrites on (111) Platinum via an Iron Oxide Interlayer

Marshall Frye[1], Jonathan Chin[1], Nicholas A. Parker[2], Steven E. Zeltmann[3], Matthew R. Barone[2,3], Darrell G. Schlom,[2,3,4,5] Lauren M. Garten[1*]

Affiliations:

1. School of Materials Science and Engineering, Georgia Institute of Technology, Atlanta, Georgia 30332, USA

2. Department of Materials Science and Engineering, Cornell University, Ithaca, New York 14853, USA

3. Platform for the Accelerated Realization, Analysis, and Discovery of Interface Materials (PARADIM), Cornell University, Ithaca, New York 14853, USA

4. Kavli Institute at Cornell for Nanoscale Science, Ithaca, New York 14853, USA

5. Leibniz-Institut für Kristallzüchtung, Max-Born-Str. 2, Berlin 12489, Germany

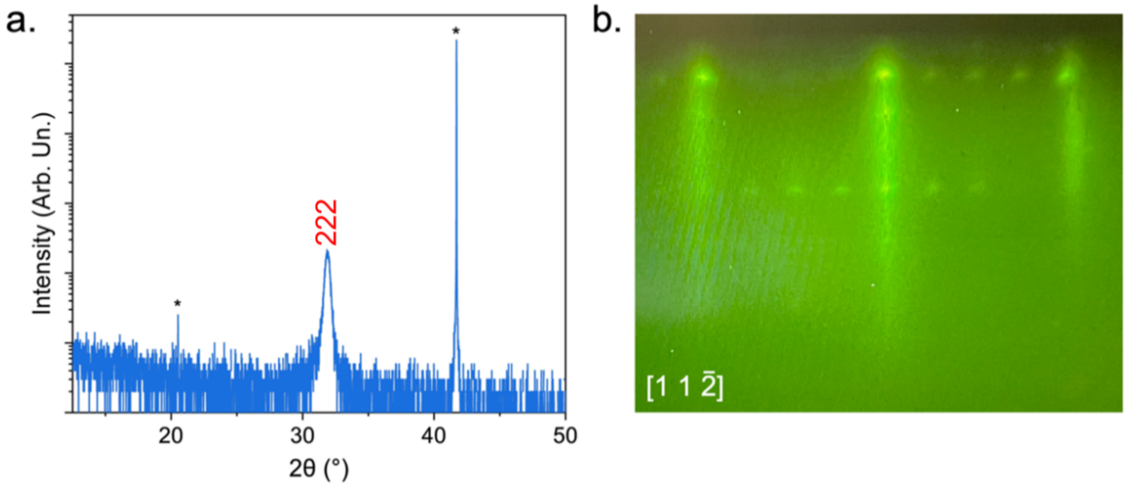

Figure S1a. X-ray diffraction and **b.** RHEED of the bixbyite phase of $ScFeO_3$ grown on Al_2O_3. The peaks denoted by * are attributed to Al_2O_3 and the peak denoted by 222 is attributed to bixbyite $ScFeO_3$ (PDF card #01-083-8521)[33]

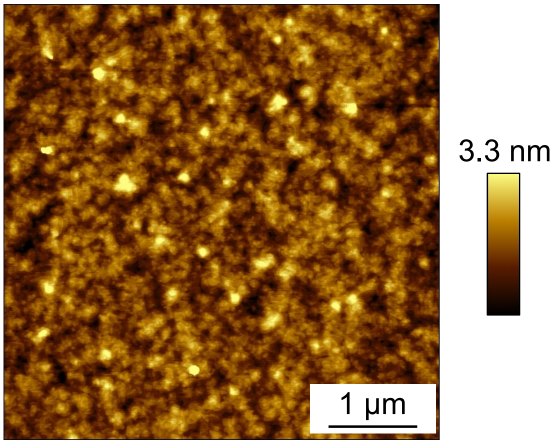

Figure S2. 5 x 5 µm AFM scan of $ScFeO_3$ grown on Al_2O_3. The average surface roughness (R_a) across the image is 0.5 nm.

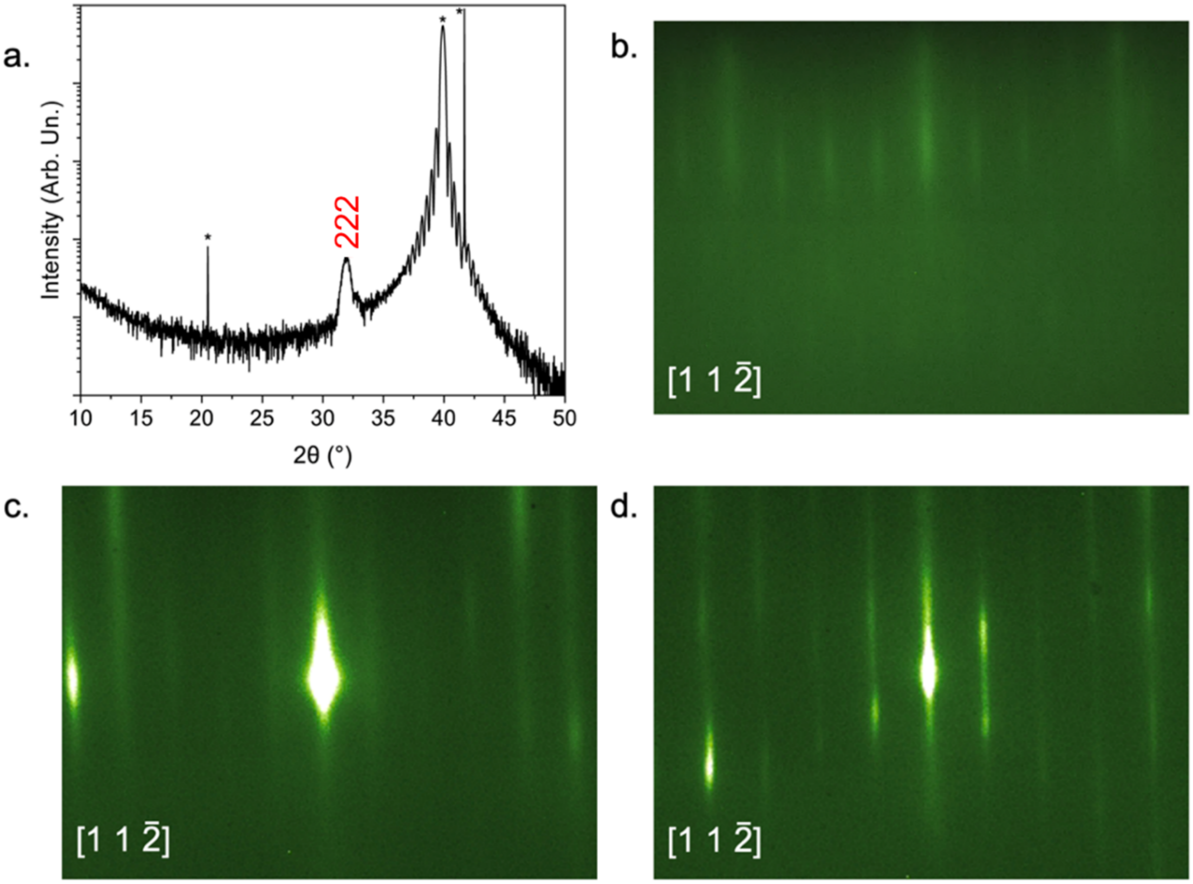

Figure S3a. XRD of a $ScFeO_3$ film deposited at 900 °C on (111) Pt and **b.** the corresponding RHEED pattern. XRD peak labelled with 222 are fit to the bixbyite phase of $ScFeO_3$ and peaks labelled with * are attributed to the (111) Pt and (0001) Al_2O_3. RHEED of $ScFeO_3$ films deposited on (111) Pt at **c.** 700 °C and **d.** 1100 °C.

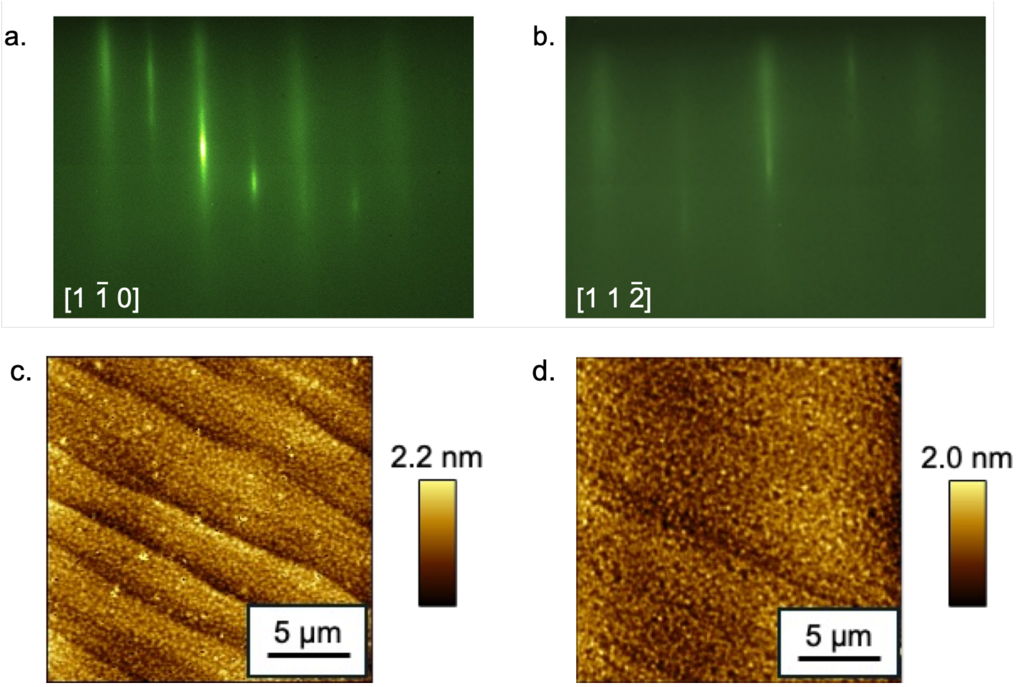

Figure S4. RHEED along the **a.** $[1\bar{1}0]$ and **b.** $[11\bar{2}]$ of the (111) wüstite structure after oxidation at 600 °C. Representative 20 x 20 μm AFM images of the **c.** Pt substrate and d**.** the iron-oxide interlayer.

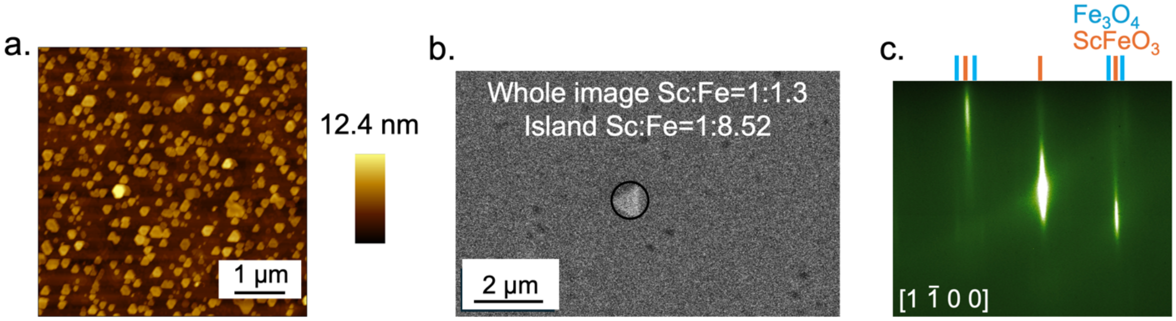

Figure S5a. AFM image of a h-$ScFeO_3$ film on (111) Pt**. b.** SEM micrograph of a representative $ScFeO_3$ film deposited on platinum. Energy dispersive X-ray spectroscopy (EDS) of the island (circled in black) indicates that the faceted islands are composed primarily of an iron oxide. **c.** RHEED of an h-$ScFeO_3$ film along the $ScFeO_3$ $[1\ \bar{1}\ 0\ 0]$ with an excess of iron at deposition temperature (900 °C).

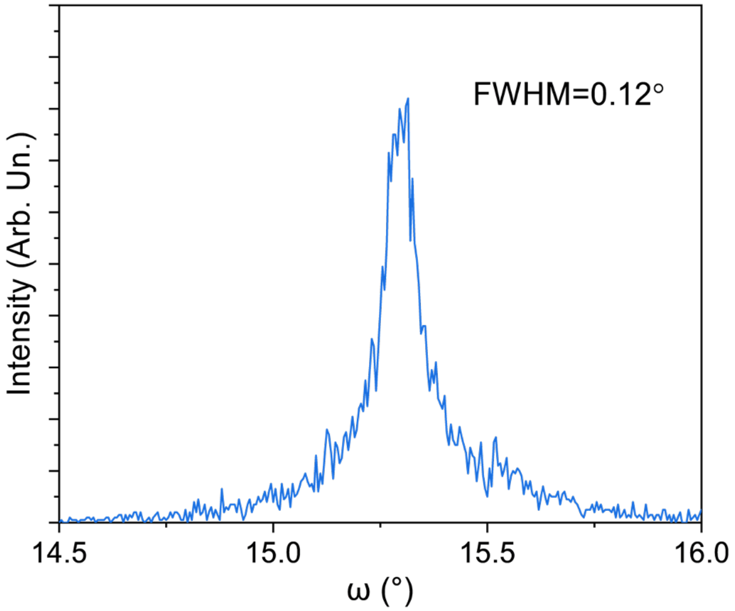

Figure S6. Rocking curve of the 0004 reflection of h-$ScFeO_3$ deposited on two layers of iron, with a measured FWHM of 0.12°.

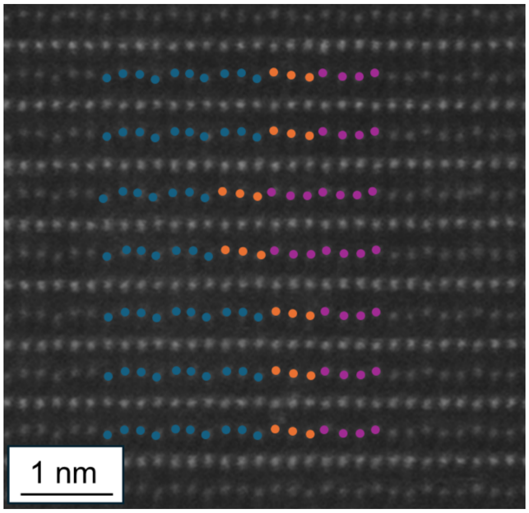

Figure S7. Scanning transmission electron micrograph of h-$ScFeO_3$ with two distinct polarization states. Blue false color contrast shows scandium atoms an up-up-down (upwards polarization vector), orange false color indicates the presumed antipolar distortion between different polarization directions (no net polarization vector), and purple false color contrast shows a down-down-up distortion (downwards polarization vector).

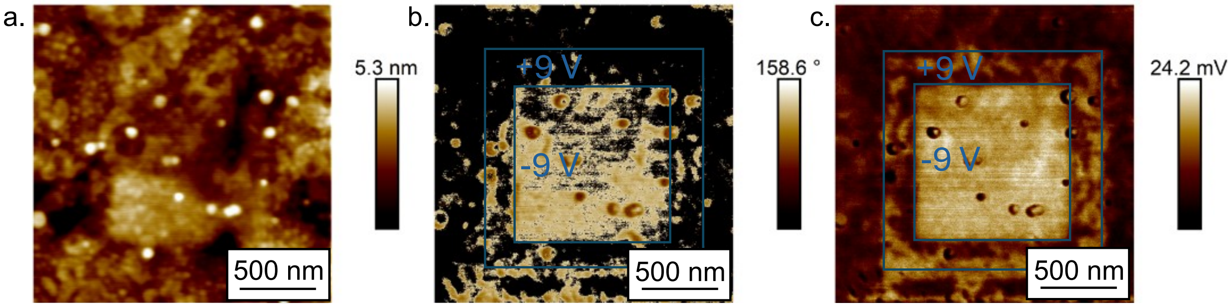

Figure S8. Vertical piezoresponse force microscopy of a 25 nm h-$ScFeO_3$ film on (111) Pt: **a.** topography, **b**. phase, and **c.** amplitude signals over a 2 µm region. An outer 1.5 µm region was poled with +9 V bias and subsequently the inner 1 µm region was poled with -9 V bias. Domain structures were measured 30 minutes after poling.

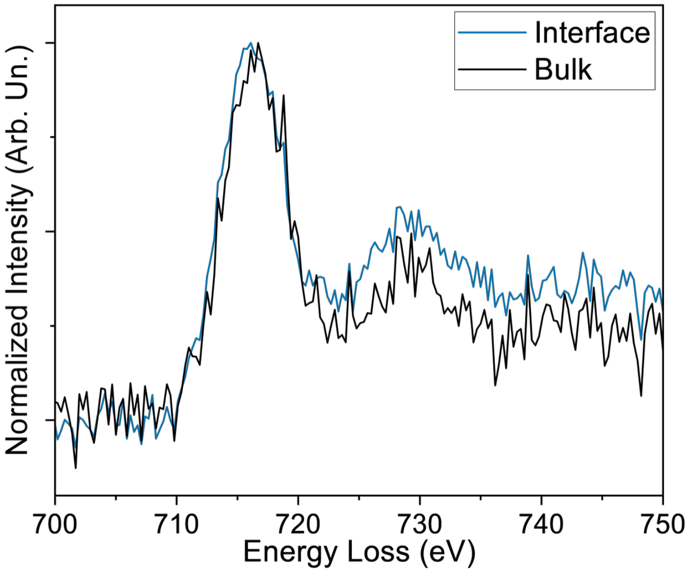

Figure S9. Fe-$L_{2,3}$ edge EELS spectra of the $ScFeO_3$ thin film deposited on an Fe_2O_3 wüstite interlayer at both the interface and bulk of the film, showing no difference in oxidation state between the iron oxide interlayer and h-$ScFeO_3$.

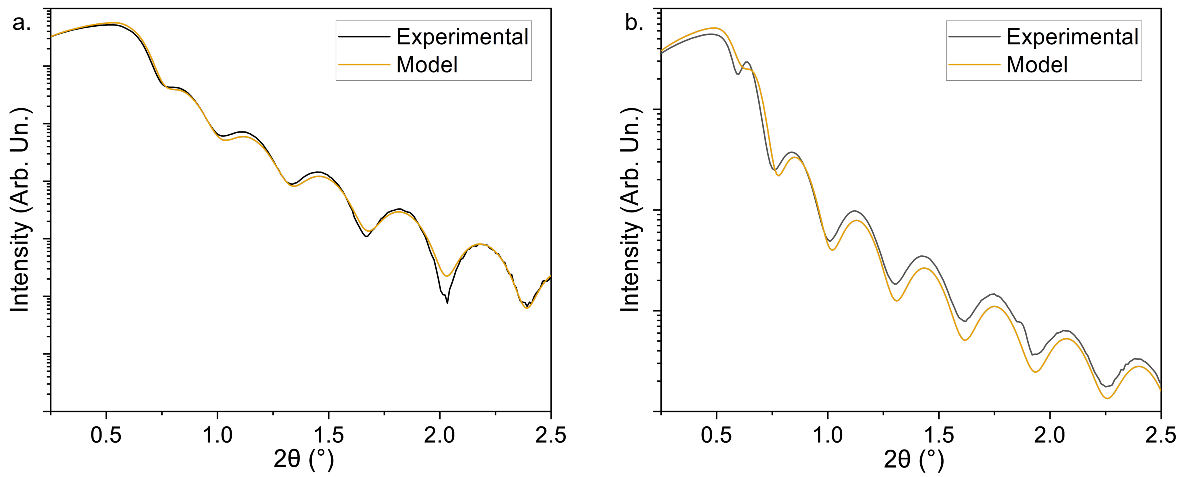

Figure S10. X-ray reflectivity measured data as well as the model used to fit the thickness, density, and roughness of the a. Fe_2O_3 and **b.** Sc_2O_3 films.

Table S1. Fit parameters used to model the Kiessig fringes of the Fe_2O_3 and Sc_2O_3 films. The R-factor is a measure of the goodness of fit, with a lower R-factor indicating a better fit.

Material	Thickness (nm)	Density (g cm^{-3})	Roughness (nm)	R-factor (%)
Fe_2O_3	23.1	5.31	1.1	1.1
Al_2O_3	∞	3.98	0.2	
Sc_2O_3	26.4	3.79	0.0	1.7
Y_2O_3 stabilized ZrO_2	∞	5.80	0.4	
